# Summary of 615 patients of chronic myeloid leukemia in Shanghai from 2001 to 2006

**DOI:** 10.1186/1756-9966-29-20

**Published:** 2010-03-03

**Authors:** Ai-Hua Wang, Yan-Yan Wang, Yu Yao, Zi-Zhen Xu, Li Zhou, Li Wang, Li Zhang, Yu Chen, Zhi-Xiang Shen, Jiong Hu, Jun-Min Li

**Affiliations:** 1 Department of Hematology, Ruijin Hospital, Shanghai Institute of Hematology, Shanghai Jiao-Tong University School of Medicine, Shanghai 200025, China

## Abstract

**Background:**

To retrospectively review the incidence, treatment efficacy, we followed up newly diagnosed chronic myelogenous leukemia (CML) patients residing in Shanghai during 2001-2006.

**Methods:**

All eligible cases were reviewed with the data of efficacy responses as well as overall survival (OS) and progression-free survival (PFS) time.

**Results:**

A total of 615 cases entered the study. CML mainly afflicted those aged 40-60 years old and was slightly more frequent in males than females. More than 85% of the patients were in chronic phase (CP) when diagnosed. All patients were divided into four groups based on the main regimens - hydroxyurea, interferon alpha (IFN-α), imatinib, and hemopoietic stem cell transplantation (HSCT). With the median follow-up of 18 months, imatinib treatment induced 92.2% complete hematologic responses, and 64.3% complete cytogenetic responses among CML-CP patients. Overall the therapeutic efficacy in the imatinib group was higher than that in the hydroxyurea or IFN-α group. Meanwhile, in the imatinib group, all response rates of patients in CP were significantly greater than that in accelerated or blastic crisis phase. The patients treated with imatinib also showed the most promising results regarding OS and PFS. Patients receiving HSCT decreased markedly in number with the introduction of imatinib.

**Conclusions:**

The number of new patients arising in Shanghai increased from 2001 to 2006. There were still patients receiving hydroxyurea and IFN-α. As the first-line regime for CML, imatinib was less administered in Shanghai before, but has received considerable development and great responses since 2003.

## Introduction

Chronic myeloid leukemia (CML) is a clonal myeloproliferative disorder associated with chromosomal translocation between chromosomes 9 and 22, which forms a fusion gene of *BCR-ABL *encoding BCR-ABL fusion protein. The excessive tyrosine kinase activity of this fusion protein activates multiple signal transduction pathways, which leads to malignant transformation [1,2].

Previous therapies for CML consisted of hemopoietic stem cells transplantation (HSCT), interferon alpha (IFN-α)-based treatment, and simple cell reduction treatment with hydroxyurea (HU). Diagnostic and therapeutic strategies for CML have progressed rapidly since the first clinical trial of targeted tyrosine kinase inhibitor imatinib mesylate (STI571, Glivec or Gleevec; Novartis Pharma) was conducted in CML patients in 1998. Currently, imatinib is considered as the first line treatment regimen for CML [3]. Recently, two additional novel kinase inhibitors, dasatinib (BMS354825; Sprycel; Bristol-Myers Squibb) [4] and nilotinib (AMN107, nilotinib; Novartis Pharma) [5], have become available as treatment options for patients who have developed resistance or those who have shown intolerance to imatinib.

We retrospectively reviewed 615 primary CML patients administered in Shanghai from 2001 to 2006 in order to evaluate diagnostic and treatment selection criteria and treatment outcomes for CML.

## Materials and methods

This was a retrospective review of local patients initially diagnosed with any stage of CML during the period January 1, 2001 to December 31, 2006. All patients whose records were reviewed were registered with the Shanghai Municipal Center for Disease Control, and validated by one of the 21 hospitals in Shanghai participating in the study. The diagnosis was confirmed by bone marrow biopsy, chromosomal and fusion gene examination.

Medical records for all patients were reviewed retrospectively with the follow-up ending on December 31^st^, 2007. Demographic data, symptoms, diagnosis, treatment, and prognosis data were collected from clinic data, written correspondence, and personal interviews.

Hematological response was defined as complete hematological response (CHR) consisting of white blood cell count <10 × 10^9^/L, platelet count <450 × 10^9^/L, with no immature granulocytes visible in peripheral blood, peripheral blood basophilic granulocyte <5%, and no extramedullary infiltration. Cytogenetic response was determined by the percentage of cells in metaphase that were positive for the Ph chromosome in bone marrow. Cytogenetic responses, based on analysis of 20 cells in metaphase, were categorized as complete (CCyR, no cells positive for the Ph chromosome) or partial (1 to 35 percent of cells positive for the Ph chromosome). Major cytogenetic response (MCyR) was defined as the combined rate of PCyR + CCyR.

Overall survival time (OS) was calculated from the date of diagnosis to the date of death or last follow-up. Progression-free survival (PFS) was measured from the acquisition of remission to the date of progression or last follow-up. Progression included the progression of CML from chronic phase (CP) into accelerated phase (AP) or blastic crisis (BC), or loss of CHR, MCyR, and CMoR. All safety evaluations were based on National Cancer Institute Common Toxicity Criteria [6].

### Statistical Analysis

Inter-group medians were compared with rank sum test and inter-group ratios with chi-square test and Fisher's exact test. The survival analysis was performed with Kaplan-Meier curve, and the survival rate and covariables were analyzed with Log-Rank test. All statistical analysis was assisted with SAS 9.0 (Cary, NC).

## Results

### Characteristics of the Patients Enrolled

A total of 615 patients were enrolled between January 1^st^, 2001 and December 31^st^, 2006. There were 325 males (52.8%) and 290 females (47.2%) with the median age of 49.5 (14-88) years old and a median follow-up time of 41 (1-78) months.

**Figure 1:**
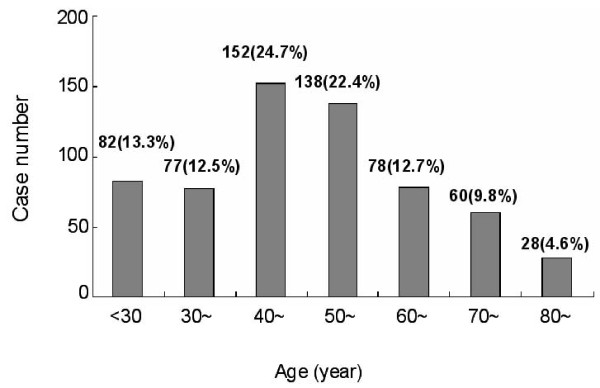
**Age Distribution of CML Incidence in the Total Population**.

The number of patients identified generally increased annually (2001, 72 patients; 2002, 68 patients; 2003, 99 patients; 2004, 113 patients; 2005, 123 patients; and 2006, 140 patients). The age distribution of CML patients was listed in Figure 1. The patients presented a wide range of ages; however, high incidence was observed in the age of 40-50 and 50-60 years old which accounted for 24.7% (n = 152) and 22.4% (n = 138) patients, respectively. The majority of patients (86.5%; n = 532) were in the chronic phase (CP) at initial diagnosis. There were 37 patients who presented in the accelerated phase (AP) (6.0%) and 46 patients in the blastic crisis (7.5%).

### Related Factors of CML Incidence

Past medical history was significant for radiation exposure in four patients, among whom one was a radiologist. Three patients received radiotherapy because of other solid tumors with intervals of 6, 38, and 61 months between the radiotherapy and the onset of CML, respectively. Exposure to chemicals (paint, gasoline, and plastic) was documented in 21 patients, among whom ten were related to the working environment; five had new apartments decorated within one year before diagnosis, and six received regular chemotherapy due to other solid tumors. Family history of hematologic disorders was identified in eight patients, including four patients of lymphoma, two patients of acute leukemia, one patient of multiple myeloma, and one patient of aplastic anemia.

### Therapeutic Regimes

In this study, 69 patients could not be followed due to various reasons, such as lose of contact or lack of clinical data. Data from the remaining 546 patients was included in the statistical evaluation.

The CML patients in Shanghai received the treatment of HU, IFN-α with/without Ara-C, imatinib, HSCT, chemotherapy, and traditional Chinese medicine. HU was still routinely used for treating almost all phases of CML, especially in patients in CP (94.1%; n = 514). IFN-α with/without Ara-C was also widely used in almost 74.2% (n = 405) of the patients. Imatinib, which has been the first line treatment for CML, was used in less than half of the patients in Shanghai because of its high cost (41.9%; n = 229). Both chemotherapy (23.6%; n = 129) and traditional Chinese medicine (18.7%; n = 102) were adjuvant therapies and were administered in combination. Chemotherapy was usually employed in two phases, the hypercellular phase and the disease progression phase, based on the type of BC (acute non-lymoblastic or acute lymphoblastic leukemia). The most common chemotherapy used were homoharringtonine (HHT), mitoxantrone (MTN), daunorubicin (DNR), arabinosylcytosine (Ara-C), and arsenic trioxide (As_2_O_3_). Among the 28 patients who underwent HSCT, 25 received allogeneic related transplantation. The oldest patient receiving transplantation was 57 years old, and the median time prior to transplantation was 7.5 (2-36) months.

### Comparison of Efficacy

Four major treatment regimes, including HU, IFN-α with/without Ara-C, imatinib, and HSCT, were evaluated in this study. The base-line characteristics of the patients were listed in Table 1. It shows that the efficacy of current treatment regimens is still unsatisfactory for both AP and BC patients. Thus, treatment efficacy was evaluated in CML-CP patients only (Table 2). On the basis of the median follow-up of 18 months, CHR, MCyR, and CCyR were achieved in 92.2%, 75.3%, and 64.3% of CML-CP patients, respectively, in the imatinib group. Rates of all measures of efficacy were substantially higher than those observed in patients who received either HU or IFN-α with/without Ara-C (*P *< 0.0001). However, no significant difference was found between the imatinib and HSCT groups. The median interval to CHR was 1.5 months in the imatinib group, 3 months in the IFN-α group, and 5 months in the HU group. The median time to CCyR was 9 months in the imatinib group, whereas it was 24 months in the IFN-α group. No cytogenetic response could be achieved in the HU group.

**Table 1:** Characteristic of the patients of four major treatment regimes

	HU	IFN-α(+Ara-C)	Imatinib	HSCT
Evaluable cases, no.	78^a^	203^b^	217^c^	28
Age, y				
Median	63	52	45.5	35
Range	23-88	19-87	14-81	19-57
Male, no. (%)	43(55.1)	108(53.2)	118(54.4)	15(53.6)
Female, no. (%)	35(44.9)	95(46.8)	99(45.6)	13(46.4)
ECOG, no. (%)				
0	62(79.5)	168(82.8)	175(80.7)	24(85.7)
1	13(16.7)	31(15.2)	35(16.1)	3(10.7)
2	3(3.8)	4(2.0)	7(3.2)	1(3.6)
Stage, no. (%)				
CP	70(89.7)	184(90.7)	154(71.0)	21(75.0)
AP	6(7.7)	12(5.9)	25(11.5)	4(14.3)
BC	2(2.6)	7(3.4)	38(17.5)	3(10.7)
Interval since diagnosis, mo				
Median	0.5	28	13	7.5
Range	0-2	0-96	0-116	2-36
White-cell count (× 10^9^/L)				
Median	25.6	31.2	28.9	21.2
Range	2.2-667	7.5-540	11.2-760	9.0-350
Hemoglobin (× g/L)				
Median	120	123	115	128
Range	68-177	56-170	66-188	70-175
Platelet count (× 10^9^/L)				
Median	345	485	520	398
Range	25-2520	21-3540	9-7050	45-2950
Peripheral-blood blasts, % (Range)				
CP	5(0-12)	4.5(0-14)	3(0-11)	4(0-9)
AP	7(2-21)	9(0-22)	4(0-29)	12(5-19)
BC	38(21-55)	36(15-60)	33(18-80)	34(15-53)
Peripheral-blood basophils, % (Range)				
CP	3(0-32)	5(0-36)	6(0-23)	4(0-20)
AP	4(0-15)	5(0-10)	3(0-11)	5(1-9)
BC	7(5-9)	4(0-12)	6(0-18)	9(3-15)
Splenomegaly, no. (%)				
Any splenomegaly	21(26.9)	61(30.0)	75(34.6)	3(10.7)
At least 10 cm	8(10.3)	28(13.8)	32(14.7)	1(3.6)

CP = chronic phase, AP = accelerated phase, BC = blast crisis, HU = hydroxyurea, HSCT = hematopoietic stem cell transplant.

^a ^On monotherapy of HU;

^b ^On IFN-α(+Ara-C) without further imatinib or HSCT;

^c ^on imatinib (excluding those of < 3 mo medication due to economic issues, transplantation and adverse events).

**Table 2:** Treatment Efficacy in CML-CP by Regimen

	HU	IFN(+Ara-C)	Imatinib	HSCT
	n = 70(%)	n = 184(%)	n = 154(%)	n = 21(%)
CHR n(%)	44(62.9)	139(75.5)	142(92.2)	17(81.0)
MCyR n(%)	0	37(20.1)	116(75.3)	15(71.4)
CCyR n(%)	0	29(15.8)	99(64.3)	15(71.4)
ND①	47(67.1)	43(23.4)	5(3.2)	0

CHR = complete hematologic response, MCyR = major cytogenetic response, CCyR = complete cytogenetic response.

^a^ND: without examination during the treatment.

### Comparison of overall survival (OS) and progression-free survival (PFS)

**Figure 2:**
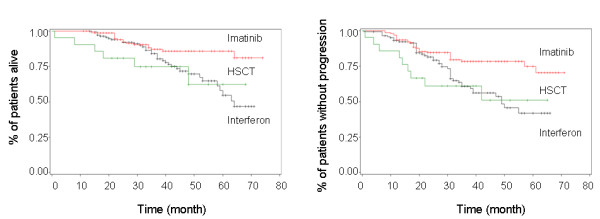
**Overall Survival (A) and Progression-free Survival (B) for CML-CP Patients by Treatment Regimen**.

OS and PFS for the major regimens (IFN-α, imatinib and HSCT) were compared in CP patients, and the results showed that both OS and PFS were significantly higher in the imatinib group compared to the IFN-α and HSCT groups (Figure 2). Estimated three-year and five-year OS rates were 88.2 ± 2.9% and 85.1 ± 3.2%, respectively, in patients who received imatinib; 74.7 ± 9.9% and 62.3 ± 14.1%, respectively, in the HSCT group; 83.8 ± 3.1% and 51.2 ± 3.4%, respectively, in the IFN-α group (*P *= 0.0075). Estimated three-year and five-year PFS rates were 79.1 ± 2.6% and 73.6 ± 3.8%, respectively, in patients who received imatinib; 61.1 ± 10.8% and 50.9 ± 12.9%, respectively, in the HSCT group; 60.1 ± 4.1% and 40.2 ± 4.9%, respectively, in the IFN-α group (*P *= 0.0021).

### Imatinib Treatment

**Figure 3:**
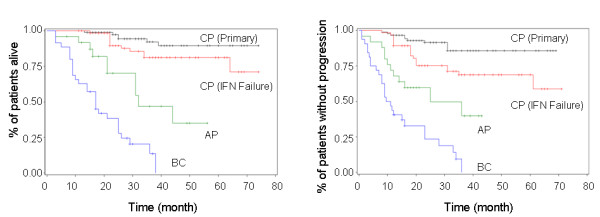
**Overall Survival (A) and Progression-free Survival (B) Among Patients Treated with Imatinib by Disease Stage**.

Among the total 229 patients treated with imatinib, 12 received the regimen for less than three months: five patients due to economic issues, five due to transplantation, and two due to adverse events. Among the total 217 evaluable patients, 114 received imatinib treatment as primary therapy and 103 had failed previous IFN-α treatment. The median time from diagnosis to imatinib treatment was 28 (4-65) months in the IFN-α failure group. Treatment efficacy (Table 3), OS and PFS (Figure 3) of imatinib were evaluated based on the stage of the disease. With the median treatment time of 18 months (range 4-61), the rates of CHR, MCyR, and CCyR were significantly higher in CP patients than those in AP and BC patients. Imatinib treatment as primary therapy was more efficient than those in patients who had failed IFN-α. Estimated three-year OS rate and PFS rate were 92.2 ± 3.4% and 85.8 ± 4.3%, respectively, in patients with CML-CP who received imatinib as primary therapy; 81.3 ± 5.4% and 68.7 ± 6.3%, respectively, in CML-CP patients who had failed IFN-α; 46.8 ± 13.0% and 39.8 ± 13.2%, respectively, in AP patients and 19.6 ± 7.4% and 10.1 ± 6.5%, respectively, in BC patients (*P *< 0.0001 and *P *< 0.0001, respectively, for OS and PFS).

**Table 3:** Efficacy Evaluation of Imatinib in CML Patients by Disease Stage

	CP		AP	BC	*P *value
		
	Primary n = 84(%)	IFN Failure n = 70(%)	n = 25(%)	n = 38(%)	
CHR	80(95.2)	62(88.6)	18(72.0)	18(47.4)	<0.0001
MCyR	71(84.5)	45(64.3)	8(32.0)	7(18.4)	<0.0001
CCyR	62(73.8)	37(52.9)	6(24.0)	4(10.5)	<0.0001

### Adverse Events

The primary side effects reported with IFN-α (+Ara-C) included fever and myalgia. A total of 25 patients (12.3%) withdrew due to grade 3 to 4 side effect. However, only two patients discontinued imatinib treatment due to intolerance (depression of bone marrow and edema), both of whom were AP and BC patients. The most common non-hematologic adverse events reported with imatinib were moderate (grade 1 or 2) nausea and vomiting (58.3%), edema (68.9%), myalgia (30%), and rash (8.2%). Grade 3/4 hematologic depression of bone marrow was reported in 17.8% of the patients.

## Discussion

The treatment of CML has undergone dramatic progress in recent years. Primary CML patients residing in Shanghai were reviewed retrospectively from 2001 to 2006, with the aim to improve the diagnosis and treatment for CML in Shanghai and to benefit the large number of patients afflicted. The number of new patients arising in Shanghai increased from 2001 to 2006. The demographic profile of CML patients in our population was similar to that described in other studies; CML mainly afflicted those 40-60 years old (47.1%), while fewer patients whose age more than 60 were affected. CML occurred slightly more in males than in females. More than 85% patients were in chronic phase of CML at diagnosis, with <15% in either AP or BC.

The etiology of CML has yet to be elucidated. Related factors were preliminarily investigated in the study; however, further investigation is needed due to lack of control data from the normal population.

HU and IFN-α were still commonly administered in Shanghai (especially to the elderly) because of financial reasons. In the population studied, 78 cases were on HU monotherapy, and 62.9% of CP patients achieved hematological response, but none of them showed cytogenetic response. IFN-α achieved lower cytogenetic response rate, probably associated with nonstandardized medication in some patients due to side effects and poor compliance. Meanwhile, chromosomes were not re-examined for about 1/4 of the patients during the period, which made it unavailable to evaluate the actual efficacy.

Imatinib was administered in a limited number of patients in Shanghai before 2003 (four in 2001 and seven in 2002) due to the high costs. With a better understanding of the regimen by both hematologists and patients, especially after the promotion offered by Glivec International Patient Assistance Program (GIPAP), the number of CML patients receiving imatinib increased dramatically from 26 patients (26.3%) in 2003, 41 (36.3%) in 2004, and 66 (53.7%) in 2005 to 85 (60.7%) in 2006. All measures of efficacy were significantly greater in patients who received imatinib as therapy for CML-CP, with successively decreasing rates of efficacy observed in those of AP and BC. Furthermore, primary therapy was more efficient than those in patients who had failed IFN-α. It may due to the longer time from initial diagnosis in the IFN-α failure group, which was about 26 months (3-56 months). Data from the International Randomized Study of Interferon alpha + Ara-C vs. STI571 in Chronic Myeloid Leukemia (IRIS) reported that the efficacy (MCyR and CCyR) of imatinib would improve further with the extension of treatment [7,8]. Imatinib also showed the most promising results in CML-CP patients with regard to OS and PFS, especially in primary patients.

Resistance to imatinib has been attributed to amplification and over-expression of the *BCR-ABL *gene, point mutation of the *BCR-ABL *gene, increased expression of other tyrosine kinases, or stem cells resistance to drugs [9-11]. Patients with resistance should be offered transplantations or new drug trials. In this study, only five were able to receive transplantations due to the lack of donors. Four patients had entered into the clinical trial of AMN107 (nilotinib) by the end of 2007. However, the majority of patients remained on imatinib in combination with chemotherapy or IFN-α due to the limited opportunities to participate in the clinical trials of new drugs in Shanghai.

Due to both limited cases and short follow-up period, the data of HSTC group was incomplete. The number of patients who received HSCT has decreased markedly with the introduction of imatinib into clinical practice since 2003. The long-term efficacy and prognosis would be evaluated with the expansion of sample size.

## Competing interests

The authors declare that they have no competing interests.

## Authors' contributions

ZXS, JML and AHW designed research; YYW, YY, ZZX, LZ, LW, LZ and YC performed research; AHW and YYW analyzed data; AHW wrote the paper, JH revised the paper. All authors read and approved the final manuscript.
